# Intrahippocampal Administration of Ibotenic Acid Induced Cholinergic Dysfunction *via* NR2A/NR2B Expression: Implications of Resveratrol against Alzheimer Disease Pathophysiology

**DOI:** 10.3389/fnmol.2016.00028

**Published:** 2016-04-26

**Authors:** Chennakesavan Karthick, Sabapathy Periyasamy, Kesavan S. Jayachandran, Muthuswamy Anusuyadevi

**Affiliations:** ^1^Molecular Gerontology Laboratory, Department of Biochemistry (DST-FIST Sponsored), Bharathidasan UniversityTiruchirappalli, India; ^2^Molecular Cardiology and Drug Discovery Laboratory, Department of Bioinformatics, Bharathidasan UniversityTiruchirappalli, India

**Keywords:** ibotenic acid, hippocampus, Alzheimer's disease, NMDAR, acetylcholine receptors, memory, resveratrol

## Abstract

Although several drugs revealed moderate amelioration of symptoms, none of them have sufficient potency to prevent or reverse the progression toward Alzheimer's disease (AD) pathology. Resveratrol (RSV), a polyphenolic compound has shown an outstanding therapeutic effect on a broad spectrum of diseases like age-associated neurodegeneration, inflammation etc. The present study was thus conducted to assess the therapeutic efficacy of RSV in ameliorating the deleterious effects of Ibotenic acid (IBO) in male Wistar rats. Stereotactic intrahippocampal administration of IBO (5 μg/μl) lesioned rats impairs cholinergic transmission, learning and memory performance that is rather related to AD and thus chosen as a suitable model to understand the drug efficacy in preventing AD pathophysiology. Since IBO is an agonist of glutamate, it is expected to exhibit an excitotoxic effect by altering glutamatergic receptors like NMDA receptor. The current study displayed significant alterations in the mRNA expression of NR2A and NR2B subunits of NMDA receptors, and further it is surprising to note that cholinergic receptors decreased in expression particularly α7-nAChR with increased m1AChR. RSV administration (20 mg/kg body weight, i.p.) significantly reduced these changes in IBO induced rats. Glutamatergic and cholinergic receptor alterations were associated with significant changes in the behavioral parameters of rats induced by IBO. While RSV improved spatial learning performance, attenuated immobility, and improvised open field activity in IBO induced rats. NR2B activation in the present study might mediate cell death through oxidative stress that form the basis of abnormal behavioral pattern in IBO induced rats. Interestingly, RSV that could efficiently encounter oxidative stress have significantly decreased stress markers viz., nitrite, PCO, and MDA levels by enhancing antioxidant status. Histopathological analysis displayed significant reduction in the hippocampal pyramidal layer thickness and live neurons in IBO induced rats, with slight pathological changes in the entorhinal cortex (EC) of rat brain, which was prevented on RSV administration. Our study thus concludes that RSV administration significantly ameliorated the deleterious effects in the IBO lesioned rat model for AD by alleviating cholinergic pathways, reducing oxidative stress and thereby improving spatial memory.

## Introduction

Glutamate is the most prominent excitatory neurotransmitter involved in almost all central nervous system (CNS) functions, especially in cortical and hippocampal regions. Nearly 70% of all excitatory synapses in the CNS of mammalian brain utilize glutamate as a neurotransmitter. Glutamate receptors, in particular, N-methyl D-aspartate (NMDA) receptors are crucial for learning and memory processes. Normal healthy individuals utilize glutamatergic neurotransmission that acts via NMDA receptors to produce a long-term potentiation (LTP). LTP refers to strengthening of synapses through repeated use and is central to the processes of learning and memory, while acetylcholine (ACh) and its receptors play a key role in both the induction and maintenance of LTP (Auerbach and Segal, 1994; Tai and Leung, 2012). ACh is released following depolarization to act on nicotinic and/or muscarinic receptors located on presynaptic cholinergic terminals, with neurotransmitter action being terminated by acetylcholinesterase (AChE). These information strongly emphasize the relationship between cholinergic and glutamatergic systems in cognitive aspects of brain function (Francis et al., 2012).

Hence, disturbances in glutamate neurotransmission may impact memory performance and has been linked with the pathophysiological processes underlying neurodegeneration and Alzheimer's disease (AD) (Wenk et al., 2006). AD is an age-associated neurodegenerative disorder characterized by loss of memory and cognitive functions, associated with chronic and a progressive neurodegeneration (Gong et al., 2004; Citron, 2010). In AD, the number of glutamatergic pyramidal neurons is significantly reduced (Francis, 1993), while in AD atrophy, dysfunction of cholinergic and glutamatergic pyramidal neurons occurs such that remaining cortical neurons receive less innervation and consequently are less likely to be depolarized by synaptic signals (Francis et al., 2012) that may be the resultant of Aβ mediated excitotoxicty. Moreover, this could be accompanied by concomitant loss of glutamatergic neurons and abnormalities of NMDA receptor expression in hippocampus of AD patients.

Therapeutics against neurodegenerative diseases like AD has long been a quest for scientist. Preventing AD and complications due to formation and accumulation of amyloid plaques particularly Aβ mediated excitotoxicity and neurodegeneration has become a mystery until today. Several drugs have shown moderate amelioration of symptoms, while none of them have sufficient potency to prevent or reverse the progression toward AD pathology. Therefore, the drug of choice that could revert homeostatic events and reduce stress levels gains the appropriation. Therefore, the current study, employs resveratrol (RSV) or 3,5,4'-trihydroxystilbene, a type of naturally occurring polyphenolic compound commonly found in red grape skin is used as a potent therapeutic target. Recent drugs focus more on preventing pathophysiology that are a consequential event of Aβ accumulation, but none could be successful in preventing one or other complications associated with Aβ toxicity. Although RSV has proved for its antioxidant properties (Marambaud et al., 2005), homeostatic effects (Moorthi et al., 2015) and more, no reports are available on the action of RSV on glumatergic and cholinergic abnormalities associated with AD conditions.

Since the present study aimed at studying the efficacy of RSV in alleviating excitotoxic effects and neurodegeneration, Ibotenic acid (IBO) a potent neurotoxin that exacerbate the symptoms and pathological variations similar to AD patients (Zola et al., 2000; Ji et al., 2009; Clark et al., 2012) has been chosen as a suitable model to understand the drug efficacy in preventing AD pathophysiology. IBO is a dynamic NMDA receptor agonist when injected into the brain of animals can elicit severe injury and even death in neurons by inducing excessive calcium influx (Zhang et al., 2013) probably through receptor activation. Abnormally high concentrations of glutamate or its analog may promote prolonged stimulation of glutamate receptors, particularly N-methyl-D-aspartate (NMDA) receptors in pathological conditions that could result in perturbed ion homeostasis, energy depletion, and the degeneration of neurons and cell death in a process called excitotoxicity (Antzoulatos and Byrne, 2004; Mattson, 2004). The current study was thus designed to determine whether RSV alleviates the pathological effects of IBO mediated neurodegeneration through susceptible receptors of major neuro-transmitter systems probably involved in learning and memory mechanisms and thereby assess the suitability of the drug in preventing AD like complications.

## Materials and methods

### Chemicals

All chemicals were procured from Sigma-Aldrich (St Louis, MO, USA) unless otherwise indicated. For RT-PCR, primers NR2A, NR2B, m1AChR, α7-nAChR, nNOS, and β-actin were obtained from Eurofins, Bangalore, India. Resveratrol were purchased from Cayman, Germany. Ibotenic acid was purchased from Abcam, Cambridge, UK. Acetylthiocholine iodide and DMSO from Hi-Media, India.

### Animals and housing

Male Wistar rats (240 ± 20 g) were purchased from Sri Venkateshwara Enterprises, Bangalore, and maintained in the animal facility at Bharathidasan University, Tiruchirappalli, India. The experiments were performed in accordance with the Laboratory Animal Care Guidelines by the Committee for the Purpose of Control and Supervision of Experiments on Animals (CPCSEA) was followed. Experimental protocol used in this study was approved by the Institutional Animal Ethical Committee (BDU/IAEC/2014/OE/06/Dt.18.03.2014). Rats were individually housed with an alternative 12-h light/12-h dark cycle. All rats were given *ad-libitum* access to food and water.

### Generation of memory deficient rat model and drug treatment

**Figure 1:**
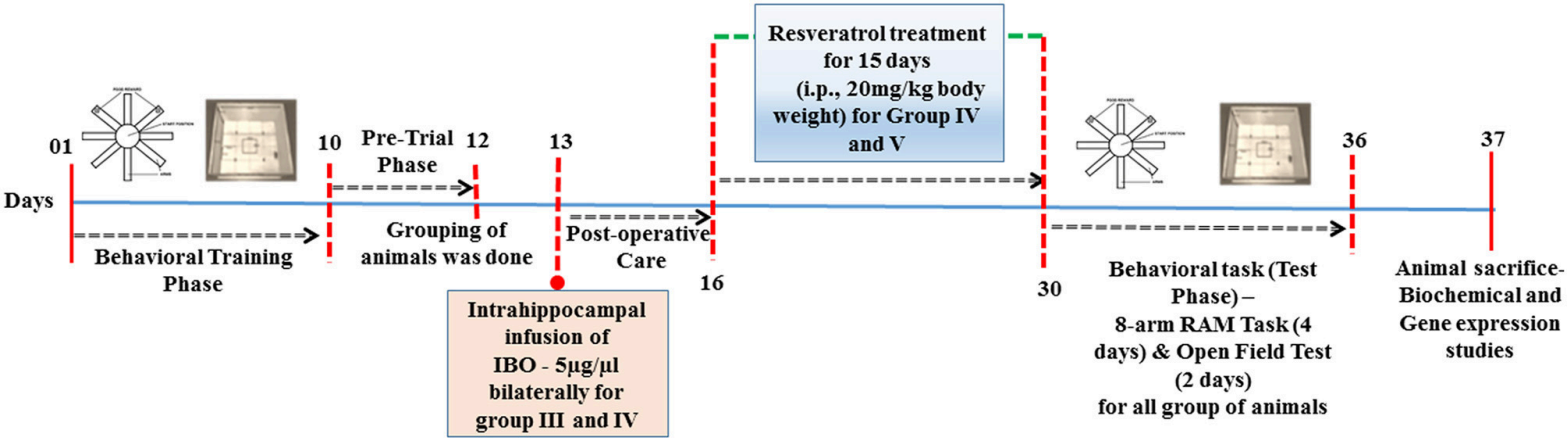
**Schematic representation of time-line for RSV treatment in IBO induced experimental animals**.

Stereotaxic surgery was performed by infusing IBO into the hippocampus of male Wistar rats. Briefly, rats were anesthetized by intraperitoneal injection of ketamine and xylazine and fixed on a stereotaxic apparatus (Ambala instrument, India). Intrahippocampal injection was made using 1.0 μl Hamilton microsyringe (Hamilton-Reno, USA) and relative to the Bregma co-ordinates for the hippocampus were –4.0, 3.0 mm mediolateral, and –3.6 mm for dorsoventral region of rats using Paxinos and Watson Atlas (sixth edition, 2007; Figure S1a). Confirmation on the infusion site was examined using trypan blue that displayed well-localized infusion to the dorsal hippocampus as shown in supplementary data, Figure S1b. Ibotenic acid was dissolved in phosphate buffered saline (PBS; pH 7.2) at a concentration of 5 μg/1 μl, then infused bilaterally into the hippocampus in a volume of 1.0 μl at a rate of 0.1 μl /min (Yu et al., 2012). After the injection, the needle was left in place for 5 min to prevent backflow. Sham-operated rats were given an injection of PBS instead of IBO solution. The skin was then sutured and the rats had fully recovered from anesthesia, and returned to their home cages. The treatment was started on post-operative day 3 with RSV (20 mg/kg body weight) for 15 days and control animals received the vehicle alone (20% DMSO). Dosage fixation was modified based on protocols described by Zhang et al. (2009) and Zhao et al. (2013). Detailed study design is provided in Figure 1.

### Experimental design

Animals were randomly assigned into six groups with seven animals each,

Group I: Normal Control or intact animals that were left undisturbed.
Group II: Sham-operated control rats-PBS infusion.
Group III: Rats injected with IBO (5 μg/μl PBS) bilaterally into hippocampus.
Group IV: Rats treated with RSV at dosage of 20 mg/kg body weight i.p., (dissolved in 20% DMSO) following IBO injection.
Group V: Rats treated with similar dose of RSV as described earlier.
Group VI: Vehicle control (20% DMSO) administered rats.

### Behavioral analysis by 8-arm radial arm maze (RAM) task

Behavioral analysis was performed as described by Olton and Samuelson (1976) with slight modifications. Rats were tested in a radial 8-arm maze for the present study. It contains eight arms numbered from 1 to 8, each arms (48 × 12 cm) extending radially from a central area (32 cm in diameter). The apparatus was placed 40 cm above the floor, and surrounded by various extra maze visual cues placed at the same position during the entire study. At the end of each arm there was a food cup that had a single 10 mg food pellet (food reward).

Prior to the performance on the maze task for behavioral analysis, the animals were restricted to partial food deprivation schedule and body weight was maintained at 80% of their free-feeding weight over a week period, with water being available *ad-libitum*. Before the actual training began (habituation), three or four rats were simultaneously placed in the RAM task and allowed to explore the maze for 5 min/day for 2 days. The food rewards were randomly scattered over the entire maze surface. On 3rd day, four of the eight arms (no. 1, 3, 5, 7) were selected and food pellets were scattered. The other four arms (no. 2, 4, 6, 8) were never baited. On the 4th day, pellets were placed in the food feeding cup at the end of the four baited arms. The number of baited arms and the spatial location of the baited arms relative to the room remained constant throughout the study. This provided the reference aspect of maze performance, where the rats were given five consecutive training trails per day for 8 days. The arm, first visited by the animal was observed.

After adaptation, to evaluate basal activity of rats in RAM task, rats were given five trials per day for 4 days during which a single food reward was placed in the food cup of the four arms assigned to be baited. Each rat was placed on the center platform facing randomly selected arms for each trial and allowed to make arm choices either of all four pellets were taken or until 5 min has elapsed. To prevent intra-maze cues (odor cues), the maze was cleaned (using 70% ethanol) between each test. An arm entry was counted when all four limbs of the rat were within an arm. Measurements were made using the number of working memory errors (entering an arm containing food, but previously entered) and reference memory errors (entering an arm that was not baited) is considered as % of correct response. The percentage of correct response was calculated by using the following formula,

$$\begin{array}{l} \%\text{ of correct response}=\text{Number of correct response}/\\ \text{ number of trials }*\text{ 100}. \end{array}$$

The latency period to enter the arm containing the food reward was recorded using a stopwatch. The time taken to consume all four baits was also recorded. All the behavioral experiments were carried out between 08.00 and 13.30 h.

### Open field test

The open field is used for measuring anxiety and exploration as well as locomotion as it has a large center arena. The open field apparatus was constructed of white plywood and measured 72 × 72 cm with 36 cm walls. The lines divided the floor into sixteen of 18 x 18 cm squares. A central square (18 × 18 cm) was drawn in the middle of the open field (Brown et al., 1999). At the beginning of the open field session, rats were placed on the right front corner square (relative to the position of the experimenter). The animals were allowed to move freely about the open field for a period of 5 min, and then returned to its home cage. The open field was cleaned with 70% ethyl alcohol and permitted to dry between tests. To assess the process of habituation to the novelty of the arena, rats were explored to the apparatus for 5 min on two consecutive days. The behavioral parameters measured was total number of grid crossings made during five trials per day for 2 days. A crossing was defined as all four paws moving out of one of the 16 squares and into another. This measure is expected to correlate highly with the distance traveled in the open field, and it reflects locomotor activity, exploratory behavior, head dipping, rearing, grooming, and anxiety (ambulations in number). Open field sessions were recorded and performance was analyzed after the testing took place.

### Tissue collection and processing

After the behavioral tests, the rats from each group (*n* = 7) were killed by cervical decapitation and brain tissues were dissected out. Following dissection, each hippocampus (*n* = 4) was weighed and rapidly homogenized in 10 volumes of ice-cold homogenizing buffer for estimating protein, nitrite levels, protein carbonyl content and antioxidant assays, the remaining tissue was rapidly frozen in liquid nitrogen and stored at −80°C for further analysis. Whole brain (*n* = 3) was removed for histopathological studies by fixing it in 10% phosphate buffered formalin at pH 7.2.

### Histopathological analysis

Formalin fixed brain tissues were processed and stained with hematoxylin and eosin (H and E) based on the protocol described by Avwioro et al. (2010) with slight modifications. Tissues, 5 mm thickness were obtained from the rat brain. They were fixed in 10% phosphate buffered formalin for 24 h and processed for embedding with paraffin wax through ascending grades of alcohol (30, 50, 70, 90, and 95%) for 1 h each, and two changes of absolute ethanol for 2 h each followed by two changes of xylene for 2 h each and two changes of paraffin wax at 60°C for 2 h each. Tissues were subsequently embedded in paraffin wax at about 60°C. Sections were cut at 10 μ thick with the microtome (Leica Instrument's). Six sections from each block were stained with hematoxylin and counter stained with 0.25% eosin. H and E stained sections were observed under light microscope and photomicrographs were captured (10x and 40x objective) throughout the stained sections for analyzing the morphological features of the hippocampus, entorhinal cortex, and amydgala of rat brain. Based on the observation, the images were selected and projected in the results.

### Extraction and quantification of RNA

Briefly, hippocampal tissue was homogenized and RNA was extracted using Trizol reagent for semi-quantitative RT-PCR analysis. Total RNA was solubilized in RNase-free H_2_O, and quantified in duplicate by measuring the optical density (OD) at 260 nm. Purity of RNA was assured by examining the absorbance ratio of OD260/OD280 using Biophotometer (Eppendorf Instrument, USA). For cDNA preparation, 2 μg of RNA was reverse transcribed using the High-Capacity cDNA Reverse Transcription Kit (Applied Biosystems, Foster City, CA), according to the manufacturer protocol. The cDNA samples were stored at −20°C until analysis. Primer specific for the genes (sequences given in Table 1) were used for PCR reactions (using Eppendorf's Master Cycler Gradient machine) with an initial denaturation at 95°C for 5 min, followed by denaturation at 95°C for 30 s, annealing (respective temperatures are given in Table 1) for 45 s and extension of 72°C for 1 min for 35 cycles for each gene (Table 1). This was followed by a final extension step at 72°C for 10 min. At first, the number of cycles was optimized in the control group of animals for all gene primer set individually. PCR products were separated on agarose gel, visualized by ethidium bromide (adding 3 μl of 10 mg/ml solution for each 100 ml of gel) staining. Respective no-RT samples for each gene were used as negative template control (data not shown). Each PCR product band was quantified for densitometry using Image J software and represented graphically.

**Table 1:** **List of primers used in this study**.

**S. No**.	**Gene**	**Primer sequence 5′–3′**	**T^a^**	**bp size**	**GenBank/Accession. No**.
1	NR2A	F = TCAGCCATTGCTGTCTTCGT	57.3	590	AF001423.1
		R = AGCGCAATTCCATAGCCTGT			
2	NR2B	F = CCACGCACACTGTCACCTAT	60.5	579	M91562.1
		R = CCTCGCTGATGTCGTACAGG			
3	m1AChR	F = AGCAGGCATGTGAATGACGG	60.6	529	XM_006230974.2
		R = GTGTCTCCTGGGACCCAAAC			
4	α7-nAChR	F = GCTGTACAAGGAGCTGGTCA	60.6	505	L31619.1
		R = GATCCCATTCTCCGTTGGGG			
5	nNOS	F = ACTGGGAGGGGAGGGATTCC	60.0	321	NM_052799.1
		R = GTCGATCGGCTGGACTTAGG			
6	β-actin	F = GCCATGTACGTAGCCATCA	57.3–60.6	375	NM_031144.3
		R = GAACCGCTCATTGCCGATAG			

*F, forward primer; R, reverse primer; T^a^, Annealing temperature; bp, base pair*.

### Protein concentration measurement

The content of protein in rat brain homogenate-hippocampus was measured by the Lowry method (Lowry et al., 1951) using bovine serum albumin as standard.

### Assay of acetylcholinesterase activity in rat brain hippocampus

Acetylcholinesterase (AChE) activity was determined according to the method of Ellman et al. (1961). Briefly, 20 mg of rat brain-hippocampus tissue was homogenized with 1.0 ml of Potassium Phosphate buffer, pH-8.0 in a Potter-Elvehjem homogenizer. 0.4 ml aliquot of homogenate was added to a cuvette containing 2.6 ml of phosphate buffer (pH-7.4) followed by the addition of 100 μl of the DTNB reagent. The absorbance was measured at 412 nm using spectrophotometer. Twenty microliters of the acetylthiocholine iodide was added and changes in absorbance per minute was calculated. The enzyme activity expressed as μmol of substrate hydrolyzed/minute/mg protein.

### Measurement of hippocampal nitrite levels

Nitrite determination in the biological material is increasingly being used as a marker for nitric oxide (NO) production. Nitrite levels were quantified by the protocol described by Green et al. (1982). Briefly, nitrite concentration in 100 μl of supernatant was measured by using a colorimetric reaction generated by 1.0 ml of Griess reagent (0.1% N-(1-naphthyl) ethylenediamine dihydrochloride and 1.0% sulfanilamide in 2.5% ortho-phosphoric acid). After 10 min of incubation at room temperature, the absorbance at 540 nm was determined and nitrite concentrations were calculated from the sodium nitrite standard curve. Results are expressed in the form of nmol/mg protein.

### Measurement of hippocampal glutathione peroxidase activity

Glutathione peroxidase destroys hydrogen peroxide with the help of reduced glutathione forming water and oxidized glutathione. The activity of glutathione peroxidase was assayed by the method of Rotruck et al. (1973). The reaction mixture consisting of 0.2 ml each of EDTA, sodium azide and reduced glutathione (80 μM), 0.4 ml of phosphate buffer, 0.7 ml of distilled water in two set of tubes, 0.1 ml rat brain-hippocampus homogenate was added to second tube and the tubes are incubated at 37°C for 5 min. Then 0.2 ml of hydrogen peroxide (25 mM) and 1.0 ml of 10% TCA is added to the first tube immediately and followed by the addition of 0.1 ml of tissue homogenate at 0 min. One microliter of 10% TCA is added to the second tube, 1 min after the addition of hydrogen peroxide, considered as 1 min. First and second set of tubes are considered as “0” min and “1” min reaction tube. The reaction mixture was centrifuged at 2500 rpm. From this, 2.0 ml of supernatant, 3.0 ml of disodium hydrogen phosphate (300 mM) and 1.0 ml of DTNB were added and the color developed was read at 420 nm and concentration of glutathione was calculated from the glutathione standard curve. The activity of GPX was expressed as μg of glutathione consumed/minute/mg protein.

### Measurement of reduced glutathione in rat brain hippocampus

Reduced glutathione in the rat brain-hippocampus was estimated according to the method described by Ellman (1959). 0.5 ml of hippocampal homogenate was precipitated with 0.5 ml of 4% sulfosalicylic acid and cold-digested at 4°C for 1 h. The tubes were centrifuged at 1200 g for 15 min at 4°C. 0.5 ml of supernatant, 2.7 ml of phosphate buffer (0.1 M, pH-8.0) and 2.0 ml of DTNB were added. The yellow color developed was read at 412 nm and glutathione concentration was calculated from the glutathione standard curve. Results were expressed as μg of reduced glutathione/mg protein.

### Determination of protein carbonyl content in hippocampus of rat brain

Two 1 mg of protein aliquots are needed for each sample to be assayed. Samples are extracted at a final concentration of 10% (w/v) TCA. The precipitates are treated with 500 μL of 0.2% DNPH or 500 μL of 2 N HCl. Samples are incubated at room temperature for 1 h with vortexing at 5-min intervals. The proteins are then precipitated by adding 55 μL of 100% TCA. The pellets are centrifuged and washed three times with 500 μL of the ethanol:ethyl acetate mixture. The pellet is then dissolved in 600 μL of 6 M guanidine hydrochloride. The carbonyl content is determined by reading the absorbance at the optimum wavelength (λ = 360–390 nm) of each sample against its appropriate blank. 2,4-dinitrophenylhydrazone protein adducts are calculated using the millimolar adsorptivity of 22.0 mM^−1^ cm^−1^ for aliphatic hydrazones. Results are reported as nmol of DNPH incorporated per mg protein (Levine et al., 1994).

### Determination of hippocampal malondialdehyde levels

In this study, we performed malondialdehyde (MDA) assay to evaluate the level of lipid peroxidation in hippocampus of control and experimental animals. The content of MDA was determined by a modified procedure of Li et al. (2010). Each hippocampus section (10 mg) was homogenized in 0.1 ml of 10 mM phosphate buffer (pH 7.4). After centrifugation at 12,000 g for 20 min, the MDA content in the supernatant was assayed by the thiobarbituric acid (TBA) reaction, and the resultant colored product was measured at 535 nm with spectrometer. The levels of TBA reactive species were expressed as mmol/mg protein. Extinction coefficient for TBARS: 1.56 × 10^5^ M^−1^ cm^−1^.

### Statistical analysis

All statistical analysis was performed using GraphPad Prism software (Version 5.0). The data were represented as mean ± SEM. Group comparison was performed using one-way ANOVA, followed by Tukey's multiple comparison tests. The criterion for statistical significance was *P* < 0.05 in all statistical evaluations; ns, non-significant.

## Results

### Resveratrol regulates NMDAR—NR2A and NR2B mRNA expression in IBO induced rats

**Figure 2:**
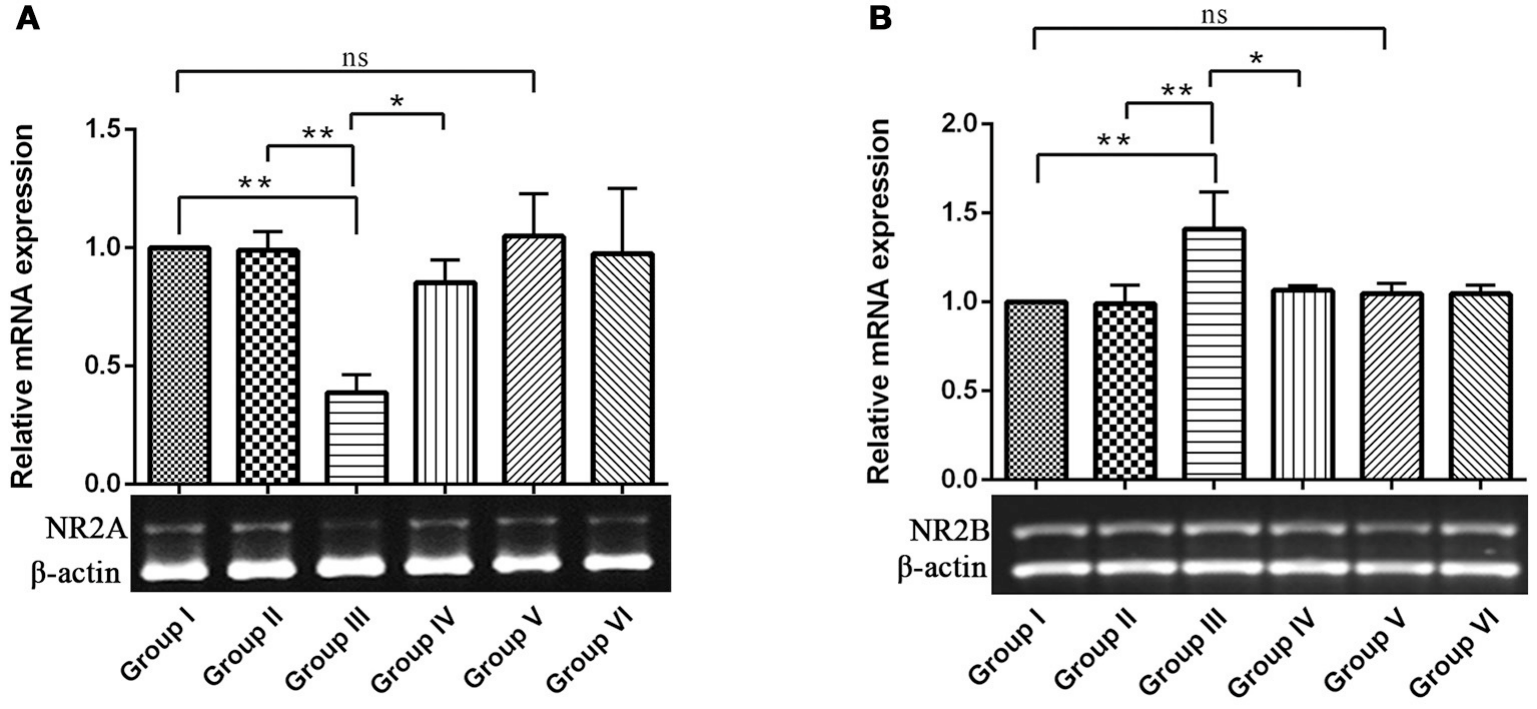
**mRNA expression of NMDA receptors (NR2A and NR2B) in control and experimental animals**. NMDARs expression levels were evaluated through semi-quantitative RT (reverse transcriptase)-PCR. **(A,B)**, Shows NR2A and NR2B mRNA expression in hippocampus of control and experimental animals with corresponding bar diagram that denotes relative mRNA expression levels which is normalized with β-actin. Values are represented as mean ± SEM (*n* = 3). Statistical significance was performed by one-way ANOVA followed by Tukey's multiple comparison test. Values are statistically significant at ^*^P < 0.05; ^**^P < 0.01; ns.

NMDAR-mediated Ca^2+^ influx into the postsynaptic cell triggers signal transduction cascades that control activity-dependent synaptic plasticity and neuronal survival (Sheng and Kim, 2002). NR2A and 2B containing NMDARs are required for both LTP and LTD, respectively. While activation of NMDARs is essential for long-lasting, activity-dependent synaptic plasticity, and involved in learning and memory (Wittmann et al., 2004). Hence, we intend to study the opposing function of NR2A and NR2B of NMDARs in hippocampus of control and experimental animals for which, we examined the relative gene expression of the NMDA receptors by semi-quantitative PCR analysis. The results depicted a significant (*P* < 0.01) down-regulation of NR2A and up-regulation of NR2B mRNA levels in IBO induced groups when compared to control groups (Figures 2A,B). Interestingly, these alteration of NR2A/NR2B mRNA levels induced by IBO was partially reversed by RSV treatment (*P* < 0.05). However, no significant changes were observed between RSV alone treated groups and respective controls (group I and VI).

### Resveratrol ameliorates morphologic abnormalities during IBO induced neurodegeneration in rat brain

**Figure 3:**
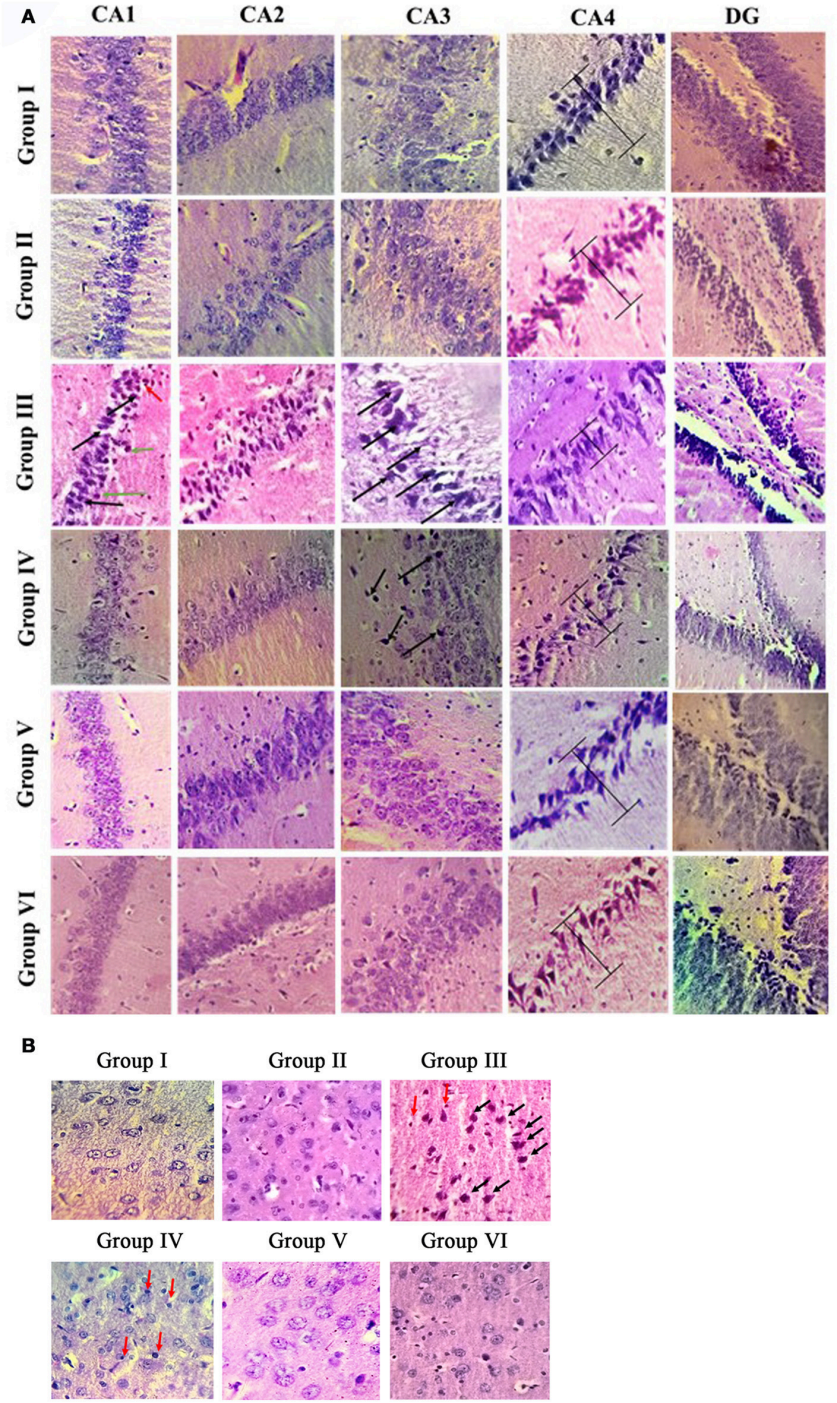
**Histopathological analysis of control and experimental group of rat brain regions:** Photomicrographs showing the effect of RSV on IBO induced *in vivo* toxicity in hippocampus of rats **(A)**. Sections were obtained from rat hippocampus 15 days after injection of IBO (*n* = 3). The morphology of the hippocampus and sub-regions are examined using H & E staining. In the IBO-model group, neuron arrangement is disrupted, severe lesions such as neuronal cell loss (black arrow), pycnotic cells (green arrow), and dead cells (red arrow) are observed in the nucleus and cytoplasm. Further prevention of degeneration through neuronal loss was attempted through RSV administration (**A**, magnification 40X). CA1, cornus ammonis 1; CA2, cornus ammonis 2; CA3, cornus ammonis 3; CA4, cornus ammonis 4; DG, Dentate gyrus. H and E staining of hippocampus associated region like entorhinal cortex **(B)** is examined for the presence of apoptotic cells, degeneration pattern etc., identifying IBO mediated pathology in associated regions from site of induction (magnification 40X).

Histologic analysis of hippocampus and entorhinal cortex in the control and experimental groups of rat brain is shown in Figures 3A,B. H & E results showed normal cellular architecture with intact cell membrane in hippocampal (Cornu ammonis—CA1, CA2, CA3, CA4, and dentate gyrus) region of control and sham control rat brain. Whereas IBO induced rat showed pathological alterations such as disorganized architecture, neuronal cell loss, and dead cells in the CA1 and CA3 region of hippocampus. In addition, pyramidal layer thickness (CA4) was reduced and cell shrinkage (CA2) were seen in IBO-induced groups when compared with control groups. Further, we intend to investigate the effect of IBO induced toxicity on the associated regions of hippocampus like entorhinal cortex and amygdala. H & E staining results exhibited pathological changes in entorhinal cortex (Figure 3B), whereas slight morphological abnormalities was observed in amygdala region (Supplementary data, Figure S2) of IBO induced group when compared with control and sham-control groups. Interestingly, RSV treated groups showed reduction in number of damaged cells and increased the number of intact neuronal cells in hippocampus and entorhinal cortex when compared to IBO-induced groups (Figure 3A). However, no specific abnormalities were observed between RSV alone treated groups and respective controls (group I and VI).

### Neuroprotective effect of RSV on mitigating acetylcholine receptor gene expression and acetylcholinesterase activity in IBO-induced rats

#### Resveratrol regulates cholinergic gene expression of experimental and control animals

**Figure 4:**
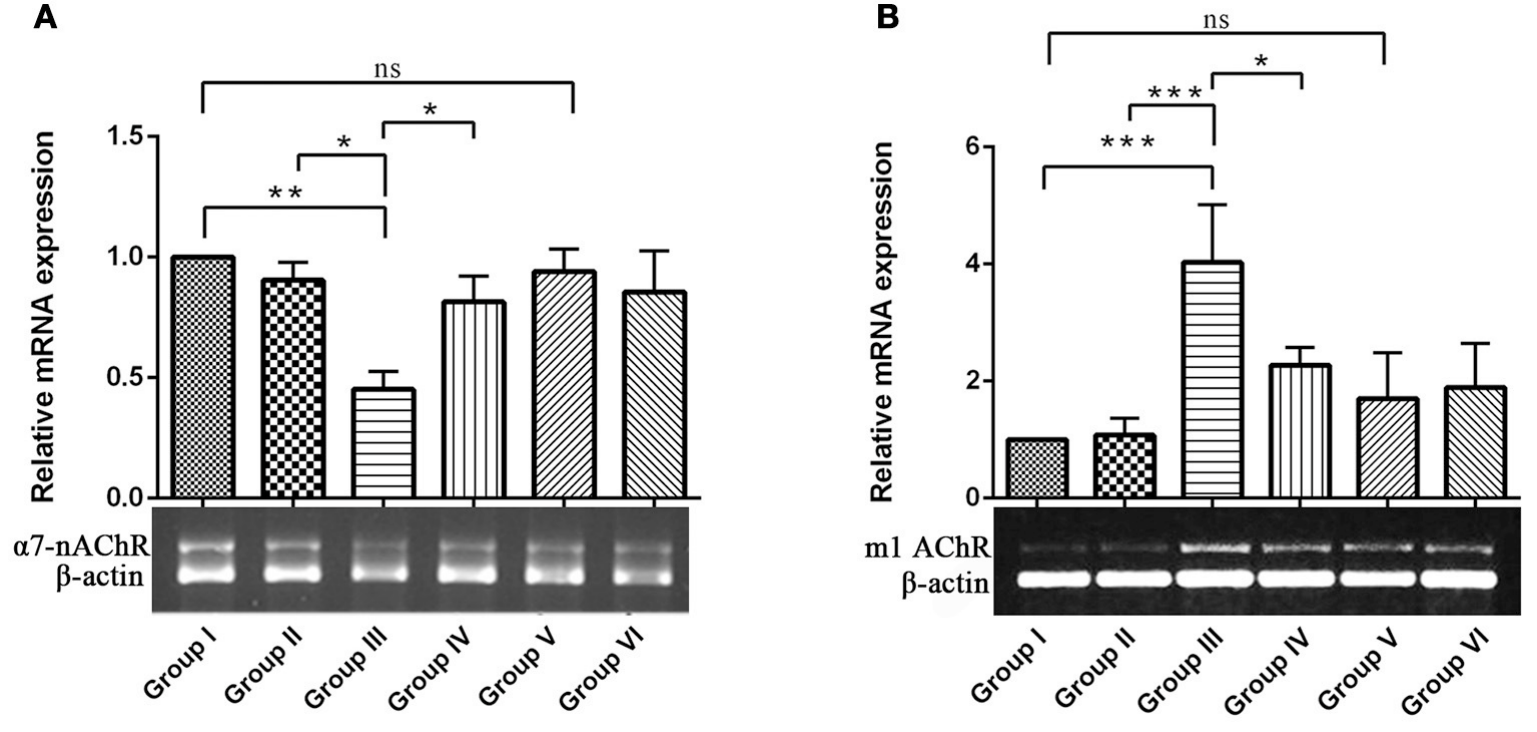
**Effects of RSV on relative mRNA expression of α7-nicotinic acetylcholine receptors and Muscarinic acetylcholine receptors 1 in IBO-induced hippocampus of rats**. **(A,B)** Shows α7-nAChR and m1AChR mRNA expression in the hippocampus of control and experimental animals with corresponding bar diagram that denotes relative mRNA expression levels which are normalized with β-actin. Values are represented as mean ± SEM (*n* = 3). Statistical significance was performed by one-way ANOVA followed by Tukey's multiple comparison test. Values are statistically significant at ^*^P < 0.05; ^**^P < 0.01; ^***^P < 0.001; ns.

To explore the mechanisms underlying the protective effects of RSV against IBO–induced cholinergic dysfunction, we examined mRNA expression of ionotrophic receptor (α7-nAChR, α7-nicotinic acetylcholine receptor) and metabotrophic receptor (m1 AChR, muscarinic acetylcholine receptor 1) in hippocampus of control and experimental animals. In Figures 4A,B, results showed that IBO-induced group exhibited significant (*P* < 0.01) down-regulation of α7-nAChR and up-regulation of m1AChR mRNA levels by *p* < 0.001 when compared to control groups. In RSV-treated groups, the expression of acetylcholine receptor, α7-nAChR and m1 AChR levels were significantly (*P* < 0.05) brought back when compared to IBO-induced groups. No significant difference was observed in the acetylcholine receptor gene expression between RSV alone treated groups and respective controls (group I and VI).

#### Acetyl cholinesterase activity

**Figure 5:**
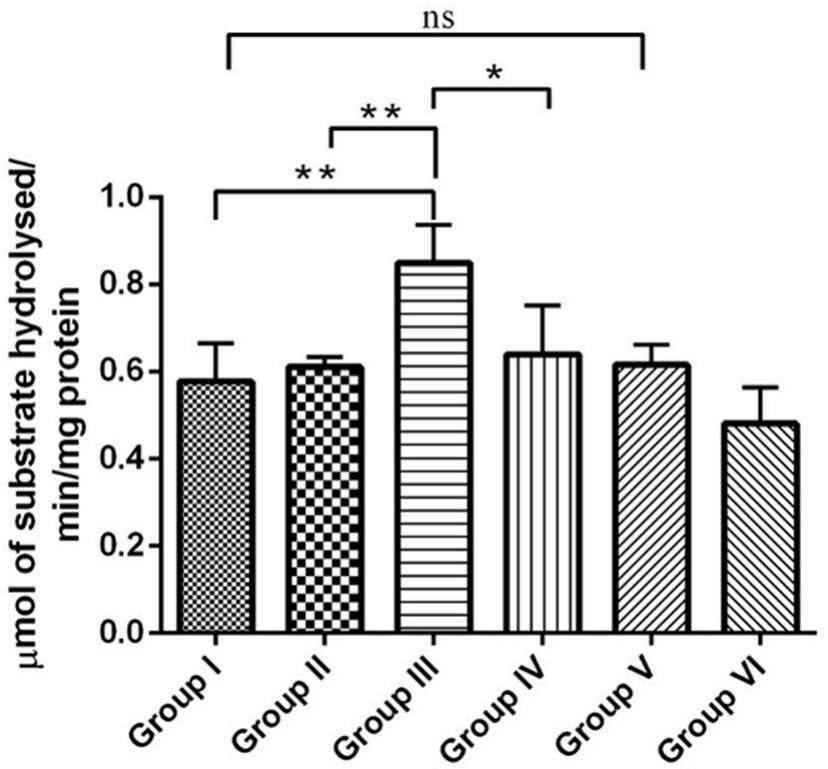
**RSV mitigates AChE activity on IBO-induced cholinergic impairments in rats**. Acetylcholinesterase activity expressed as μmols of substrate hydrolyzed/min/mg protein in the hippocampus of control and experimental animals. Values are represented as mean ± SEM (*n* = 4). Statistical significance was performed by one-way ANOVA followed by Tukey's multiple comparison test. Values are statistically significant at ^*^P < 0.05; ^**^P < 0.01; ns.

The cholinergic pathway is well-known for its role in learning and memory in the mammalian limbic system. Acetyl cholinesterase (AChE) is a key enzyme involved in cholinergic neurotransmission. Reductions in cholinergic function occurring early are well-correlated with the degree of cognitive impairments in patients with AD. In the present study, the effect of RSV on the level of neurotransmitter AChE in hippocampus of experimental groups were assessed. The enzymatic activity of AChE was significantly increased (*P* < 0.01) in the IBO-induced groups when compared with controls (Figure 5). The observed increase in the level of AChE was moderately reduced upon treatment with RSV (*P* < 0.05). However, no significant difference in the levels of AChE activity in RSV alone treated group and respective controls (group I and VI) were observed.

### Behavioral observations

#### Resveratrol enhances behavioral performance during IBO induced rats

**Figure 6:**
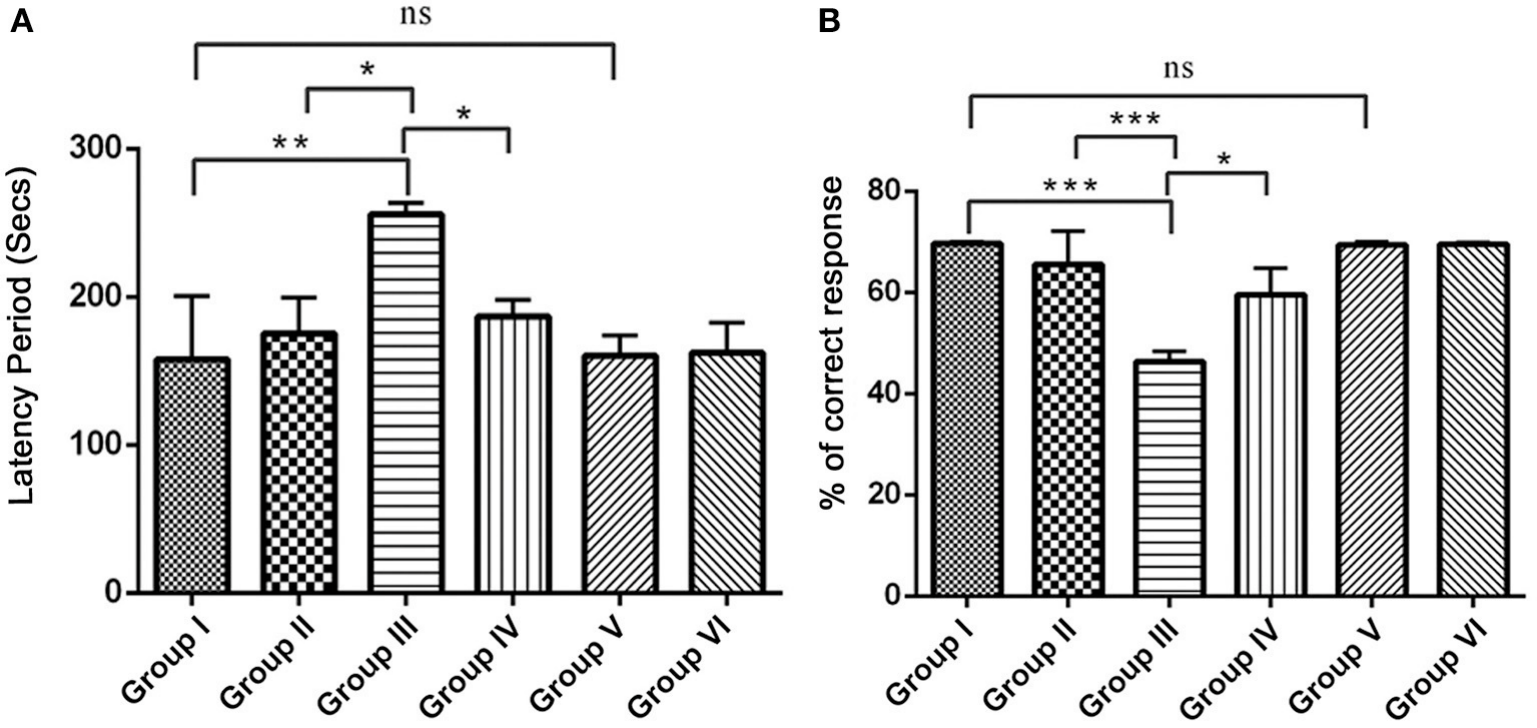
**Effect of RSV on spatial learning and memory of control and experimental rats using radial arm maze (RAM) task**. The overall memory score was calculated on the basis of both training and the test phase (*n* = 7). **(A)** Shows the comparison of Retention time (latency period) of animals on 8-arm radial arm maze (RAM) task. The bar diagram represents the retention time of acquired task, where the retention time was assessed for 4 days following the procurement of the task. **(B)** Represents % correct response as depicted by the performance of animals in 8-arm RAM baited task. The bar diagram depicts the mean percentage of correct choices made by the rats as a function of time. Each animal received five trials/day for 4 days. Values are represented as mean ± SEM. Statistical significance was performed by one-way ANOVA followed by Tukey's multiple comparison test. Values are statistically significant at ^*^P < 0.05; ^**^P < 0.01; ^***^P < 0.001; ns.

Since, IBO is a powerful excitatory neurotoxicant that may lead to the cholinergic dysfunction and memory impairments, we determined whether RSV influences positive effect on IBO induced memory defect in rats. For this, we evaluated spatial learning and memory by assessing latency period (Secs) and % correct response in 8-Arm RAM task after 15 days of treatment in experimental groups. The correct choices out of four daily trials, IBO induced groups displayed a decrease of 45%, and the latency period was increased to about 240 s when compared to control groups. This shows a significant (*P* < 0.001) impairment in hippocampal lesioned rats in way of making correct choices (both working and reference memory error). Treatment with RSV for 15 days significantly (*P* < 0.05) improved RAM performance thereby preventing spatial learning and memory defects, specifically the number of correct choices was 64.5% and the latency period was by 180–210 s was observed. There was no significant difference between RSV alone treated groups compared to respective controls (group I and VI) as shown in Figures 6A,B.

#### Open field activity

**Figure 7:**
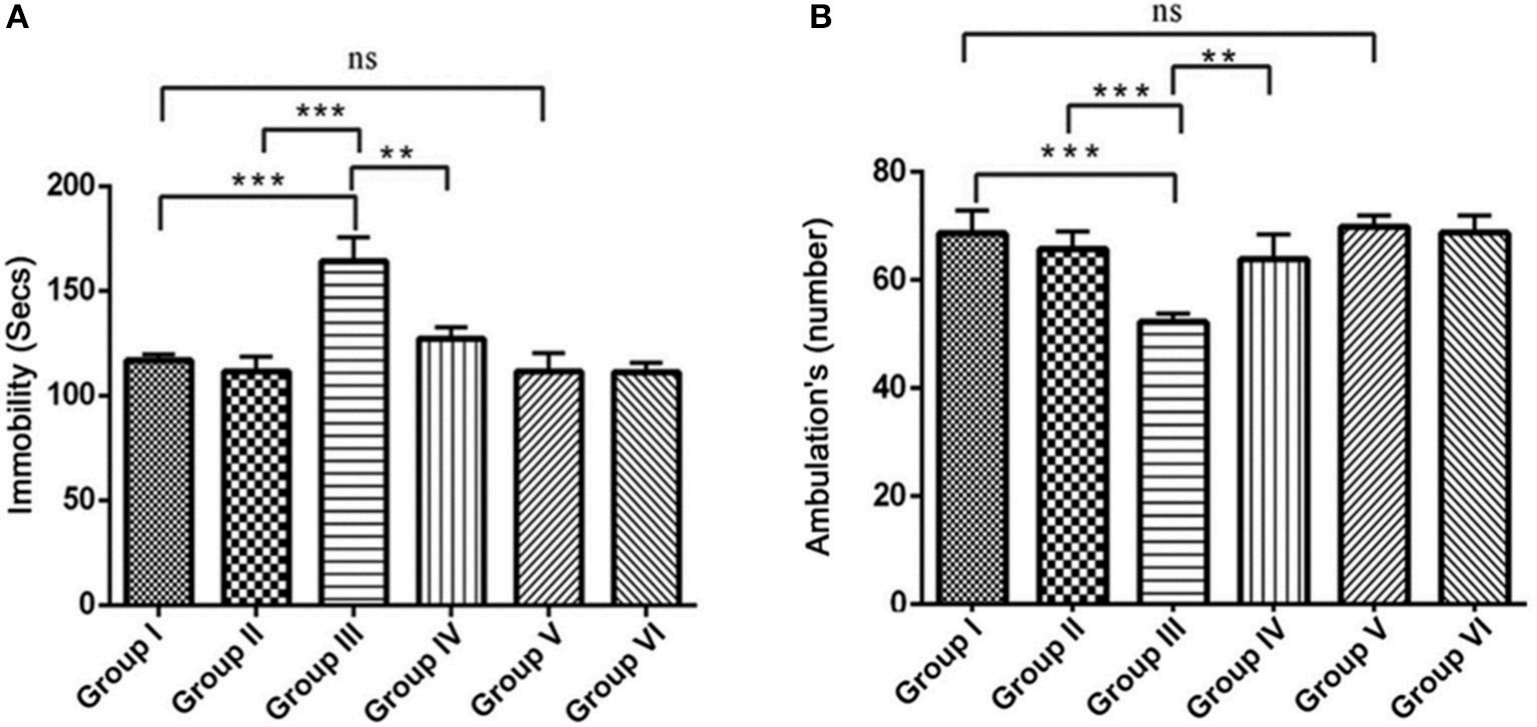
Effect of RSV on ambulation and locomotor activity of rats assessed in the open field test for the 5 min period for two successive days. Open field test activity was measured as the number grid crossing in 5 min. **(A)** Depicts the period of immobility in seconds by animals in the open field maze; **(B)** Represents ambulatory activity of the animals in the open field task. Values are represented as mean ± SEM (*n* = 7). Statistical significance was performed by one-way ANOVA followed by Tukey's multiple comparison test. Values are statistically significant at ^**^P < 0.01; ^***^P < 0.001; ns.

The open field test is conventionally designed to assess the state of anxiety, locomotor and exploratory activity. In comparison with the control groups, the IBO induced group showed increased propensity toward being immobile (164.3 ± 11.38, *P* < 0.001) and decreased number of ambulation's (51.71 ± 1.692, *P* < 0.001). When IBO induced rats treated with RSV exhibited period of immobility with a value of 135.5 ± 8.185 that depicts a corresponding decrease and significantly increased number of ambulation's (63.92 ± 4.516) when compared to that of IBO induced group (*P* < 0.01). The decreased immobility of rats in the open-field is a characteristic pattern indicating reduced anxiety, while IBO induced rats shown more head dipping compared to RSV treated group (data not shown). There was no significant difference in RSV alone treated group and respective control groups (Figures 7A,B).

### RSV reversed IBO-induced nNOS expression and nitrite production in the hippocampus of rat brain

**Figure 8:**
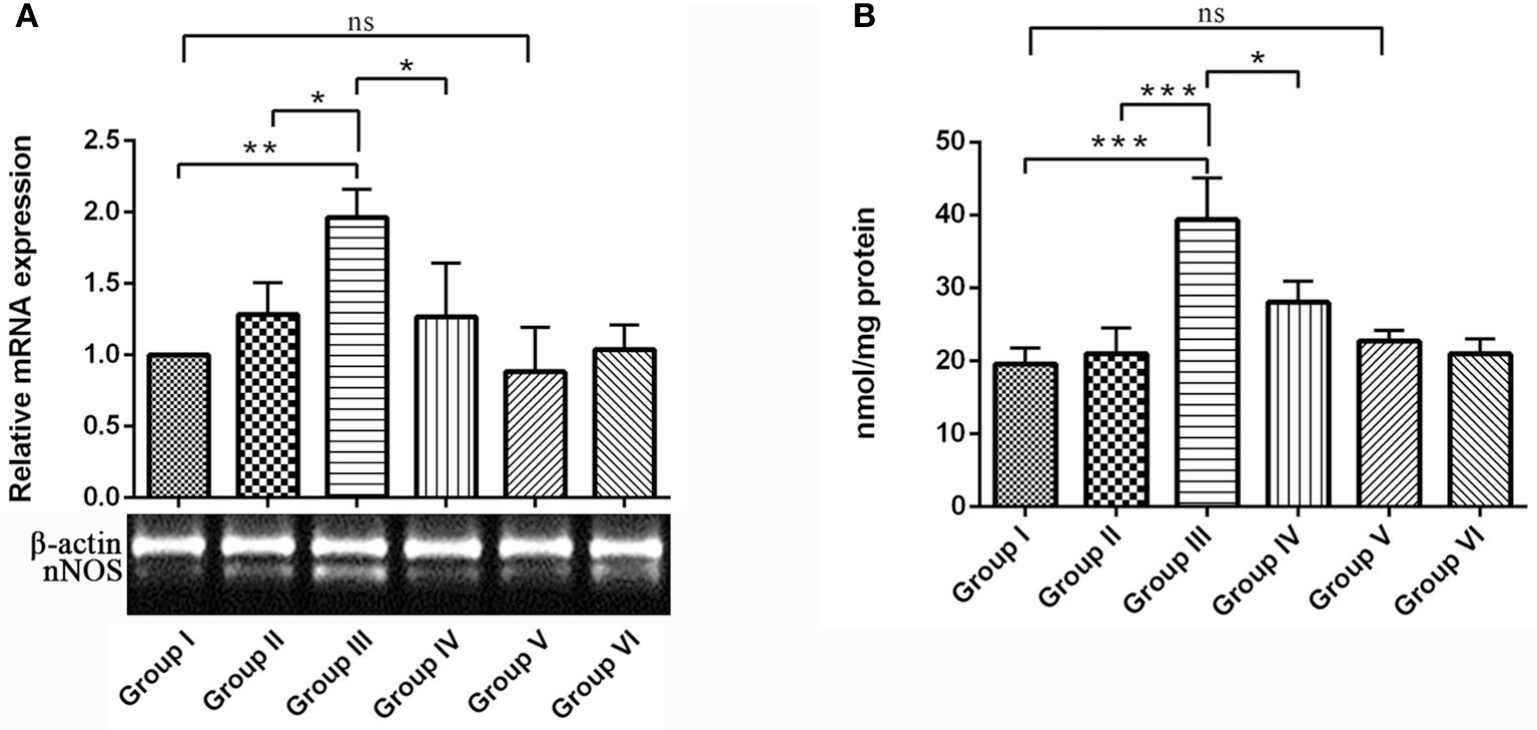
**Effect of RSV on nitrite levels and its relative mRNA expression of nNOS gene in the hippocampus of IBO lesioned rats**. Panel **(A)** denotes nNOS expression levels of control and experimental animals in representative gel image with corresponding bar diagram that indicates relative mRNA expression of nNOS normalized with β-actin (*n* = 3). Panel **(B)** Nitrite levels expressed as nmol/mg protein in the hippocampus of control and experimental animals (*n* = 4). Values are represented as mean ± SEM. Statistical significance was performed by one-way ANOVA followed by Tukey's multiple comparison test. Values are statistically significant at ^*^P < 0.05; ^**^P < 0.01; ^***^P < 0.001; ns.

Present study assessed the levels of nNOS mRNA expression and accumulation of nitrites in the hippocampus of rat brain. nNOS mRNA expression was significantly up-regulated in the IBO induced group compared with control groups (*P* < 0.01). Interestingly, RSV treatment significantly (*P* < 0.05) normalized the nNOS expression when compared to IBO treated group (Figure 8A). In the brain, the enzyme nNOS is responsible for nitric oxide production under pathological conditions, including neurodegenerative diseases. Hence we intend to study the effect of RSV on nitrite levels in IBO induced rats. Nitrite levels were significantly increased (*P* < 0.001) in the hippocampus of IBO induced group compared to control groups. IBO-induced rats treated with RSV showed significant (*P* < 0.05) reduction in the level of nitrite compared with IBO-induced group (Figure 8B). However, no significant difference between RSV alone treated group and respective controls (group I and VI) were observed.

### Effect of RSV on oxidative parameters in the hippocampus of IBO induced rats

**Figure 9:**
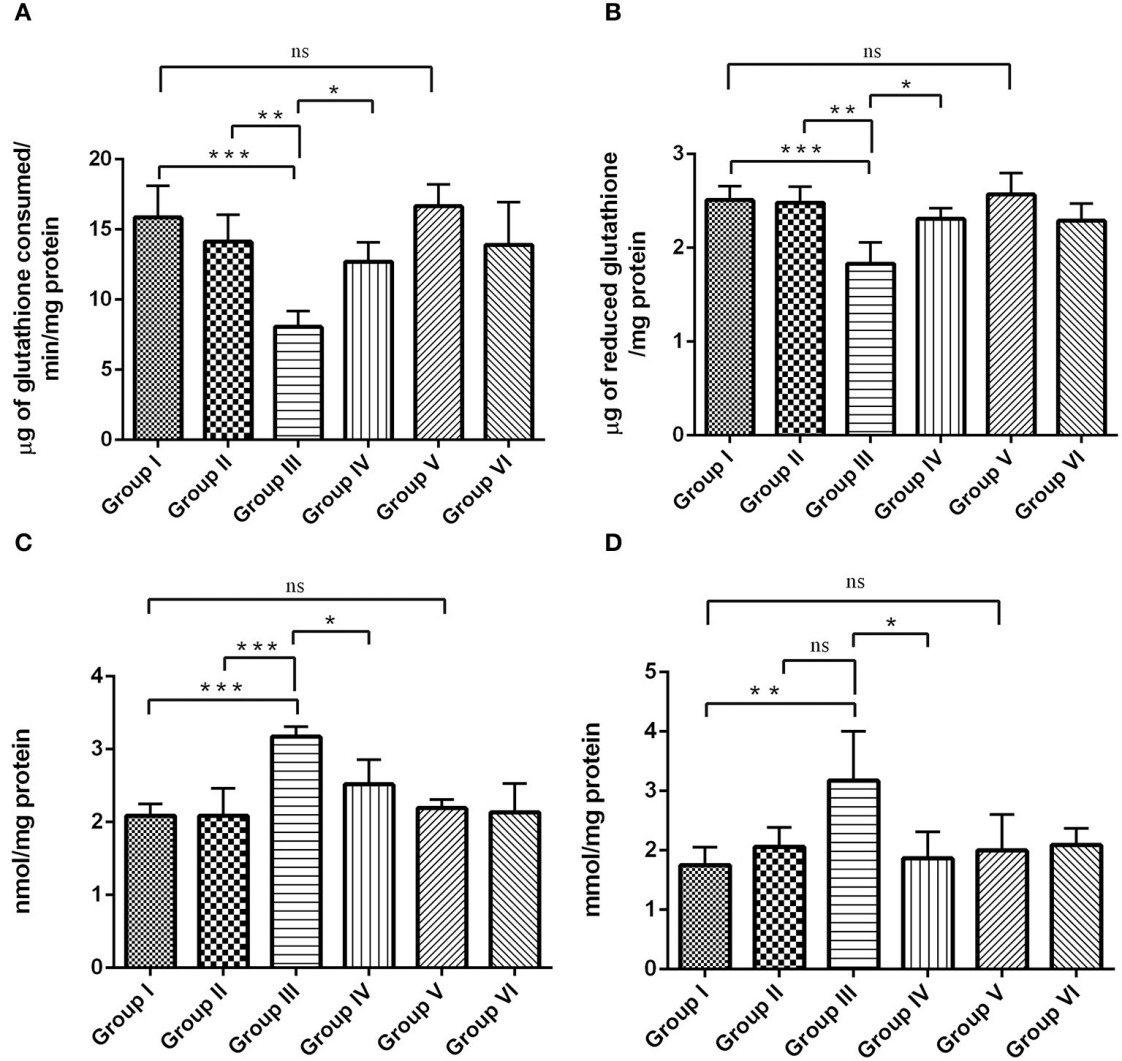
**RSV exhibits antioxidative potential by mitigating IBO-induced oxidative stress**. Antioxidant efficiency of RSV on IBO induced rats was assessed by determining enzymatic antioxidants like **(A)** glutathione peroxidase (μg of glutathione consumed/min/mg protein), **(B)** reduced glutathione levels (μg of glutathione/mg protein) and its efficacy in reducing oxidative stress was determined by evaluating **(C)** protein carbonyl content (nmol/mg protein), and **(D)** malondialdehyde levels (mmol/mg protein) in the hippocampus of control and experimental rats. Rats were exposed to IBO lesions (5 μg/μl) for 15 days and effect on these parameters assessed immediately after evaluating the behavioral performance. Values are represented as mean ± SEM (*n* = 4). Statistical significance was performed by one-way ANOVA followed by Tukey's multiple comparison test. Values are statistically significant at ^*^P < 0.05; ^**^P < 0.01; ^***^P < 0.001; ns.

Figures 9A–D, shows the status of antioxidant and oxidative parameters such as GPx, GSH, PCO, and MDA levels in the hippocampus of control and experimental groups. GPx (Figure 9A) and GSH (Figure 9B) levels were significantly (*P* < 0.001) reduced, whereas the levels of PCO (Figure 9C) and MDA (Figure 9D) was significantly elevated by *p* < 0.001 and *p* < 0.01, respectively, during IBO induction when compared to control groups. Interestingly, the adverse changes in antioxidant enzymes (GPx and GSH), PCO and MDA levels were reversed as that of control in RSV treated group (*P* < 0.05) when compared to IBO induced group. However, no significant difference in oxidative parameters was observed between RSV alone treated group and respective controls (group I and VI).

## Discussion

Evidence proved that cholinergic input to the hippocampus plays an important role in learning and memory and that degeneration of cholinergic neurons in the hippocampus may contribute to memory loss associated with neurodegenerative disease like Alzheimer's disease (AD) (Pepeu et al., 2015). Current study employs IBO, a glutamate agonist for the induction of excitotoxicity mediated cholinergic dysfunction in the regions of hippocampus to study the role of RSV in preventing neurodegeneration. Excitotoxicity is mediated by excessive and dysregulated activation of glutamate receptor (NMDA), where prolonged exposure to high or persistently increased concentration of glutamate/glutamate agonist can lead the cell expressing these receptors to death (Choi, 1994; Degos et al., 2013). Observation on the levels of hippocampal NR2B displayed a significant increase in the mRNA levels with decreased NR2A subunit of NMDA was evident in the current study (Figures 2A,B). These are the ligand binding domains of NMDA in the pyramidal neurons of the hippocampus where, NR2B involves in efficient calcium influx promoting synaptic plasticity (Nicolai et al., 2010). Current observation of increased NR2B over NR2A clearly implies that this metabotrophic channel expressed widely in response to IBO might allow increased Ca^2+^ influx, thereby mediating cell death.

In line to the above suspicion, current observation with IBO induction, mediated significant pathological changes in regions of CA1 and CA3 with increased dead cells and displayed disorganized arrangement of cells in hippocampus (Figure 3A). Increased Ca^2+^ influx through NMDA receptors could directly activate catabolic enzymes such as proteases, phospholipases, and nucleases that directly cause cell demise and tissue damage like cytoskeleton/membrane damage (Berliocchi et al., 2005). These observations well-correlate with Aβ induced toxicity that are mediated by several mechanisms, like oxidative stress, alterations in membrane potential, synaptic dysfunction, excitotoxicity through its interaction with the neurotransmitter receptors (Canevari et al., 1999; Rosales-Corral et al., 2004; Parameshwaran et al., 2008). Therefore, the present study using IBO induction in hippocampal CA3 region mimics AD condition that could facilitate efficient drug target to these aspects of neurodegeneration.

RSV administration to IBO induced rats has efficiently brought forth normalization of receptor expression viz., NR2A/NR2B levels and significantly reduced the morphological changes in the hippocampus of experimental rats as shown in Figures 2A,B, 3A,B thereby preventing excitotoxicity mediated cell death. Cellular energy stores are depleted during excitotoxicity with a decrease in levels of ATP and nicotinamide adenine dinucleotide (NAD+). NAD+ is an important energy substrate and cofactor involved in multiple metabolic reactions (Brennan et al., 2006), including glycolysis, DNA repair processes, and the function of several NAD+-dependent enzymes, such as the histone deacetylase, SIRT1 (Liu et al., 2008). Therefore, loss of SIRT1 activity is assumed to be an evident phenomenon during excitotoxicity, and thereby RSV administration expected to positively induce cellular metabolic stress response preventing energy deficits through a compensatory mechanism by activating sirtuin mediated pathways. Our previous study on RSV was found to be effective in enhancing SIRT1 levels during normal aging (Moorthi et al., 2015). Thus, it is clearly evident that RSV could have prevented further toxic effects by normalizing SIRT1.

It is noted that prolonged activation of NMDA receptor by IBO infusion might trigger enormous Ca^2+^ influx that could impact cholinergic neurons expressing specific ACh receptor subtypes. nAChRs, particularly α7-nAChR having one of the highest permeability to calcium ions and are regulated by cytoplasmic calcium levels, suggesting a complex reciprocal relationship (Jack and Wonnacott, 2015). Present study demonstrated that IBO induced rats reduced hippocampal mRNA expression of α7-nAChR that may lead to cholinergic dysfunction (Figure 4A). Levin et al. (1999) demonstrated decreased functioning of hippocampal α7 receptors that contribute to cognitive impairment by chronic systemic co-infusion of mecamylamine and IBO lesions.

Studies provide evidence that (mAChR) muscarinic receptor gene product mediates the modulation of NMDAR-mediated excitatory synaptic transmission (Marino et al., 1998). Reports also suggest that activation of m1 muscarinic ACh receptor in hippocampal interneurons decreased the function of α7-nAChRs in a calcium-dependent manner (Shen and Yake, 2009). Surprisingly, study on m1AChR levels on IBO induced rats displayed significant increase in its mRNA expression (*P* < 0.001) as seen in RT-PCR analysis (Figure 4B). Current observations clearly evidence the strategies in response to excitotoxicity by upregulating m1AChR expression with significant down regulation of nicotinic ACh receptors sensing Ca^2+^ pool inside the neuronal cells. Interestingly, RSV administration has significantly reversed the abnormalities of α7-nAChRs and m1AChR in IBO induced rats (Figures 4A,B).

Reduced expression of α7AChR during IBO induction could impact ACh levels by reducing its concentration in the synaptic cleft. Reduction in the expression of α7-AChR thereby questions the status of AChE activity during excitotoxicity in regulating ACh levels. In support of this view, it was interesting to note that AChE was found to be significantly increased (*P* < 0.01) in IBO induced rat hippocampus, which was brought back to near normal on administration of RSV (Figure 5). An increased AChE activity may have a significant role in the reduction of ACh level, in response to reduced α7-AChR. While prolonged disruption of ACh release raise the possibility of disrupting cholinergic nerve impulse transmission and thereby might delay the transmission of signals to adjacent regions of rat brain leading to gradual neurodegeneration. It is reported that IBO-induced lesions to medial septum, at the source of hippocampal branches of the forebrain cholinergic projection system, rats showed long-lasting stable impairment in reference and working memory in both spatial (place) and in associative (cue) radial maze tasks (Hodges et al., 1991).

Consistent with the previous reports, the present study demonstrated that the injection of IBO produced significant memory impairment due to disturbed synaptic plasticity and loss of spatial working memory (Figures 6A,B) that might be due to alterations in cholinergic markers as indicated by increased AChE activity reducing ACh in hippocampus. It has also been reported that modulation in NR2A gene may produce viable cells with impaired plasticity (Rolf et al., 1998) and is in line with the current observations. Current study displayed significant reduction in memory performance and locomotor activity indicating impairment in either of the excitation nerve impulse transmission modes.

Learning and memory are complex processes involving several brain regions and neuronal networks, where the hippocampus and its associated region play a critical role in generating cognitive maps. Reference memory is trial independent that is relevant for spatial cue locations while working memory is trial dependent one (Luine et al., 1998). Here, we used RAM task to assess spatial as well as working memory performance that helps study the cognitive behavior of IBO induced rats. However, during Trial 1, there was no significant difference in the response among groups, suggesting that all rats began each session of learning and memory task at a similar level. During subsequent trials, there was a significant difference (*P* < 0.001, Figure 6B) in lesioned rats, indicating the defects in the learning memory. We found that post-training of rats after IBO lesion seems to be more disruptive when compared to rat's performance before IBO induction (data not shown). This was suggested due to problems in the glutamatergic neurons leading to a deficits in place memory and episodic memory as well (Viskontas et al., 2000; Neves et al., 2008). RSV treated rats performed with an increased percent of correct response when compared to control groups (Figure 6B). Research reports provided evidence that RSV improved impaired learning and memory in neurodegenerative disease like AD, HD models or cognitive impairment induced by Scopolamine, Prenatal stress and Kainic acid (Gacar et al., 2011; Pushpalatha et al., 2013; Sahu et al., 2013).

Open field activity may be sensitive to hippocampal CA3 and mossy fiber pathways (Lim et al., 2001) and therefore increased open field activity has been used as an index of locomotor, anxiety and exploratory activity in IBO induced rats (Figure 7A). Significant reductions in open field activity were observed in RSV treated rats (*P* < 0.01), indicating better locomotor and exploratory activity, as shown in Figure 7A. As observed earlier, the present study showed that RSV normalized the expression of both α7-nAChR and m1AChR mRNA expression which could have further reduced the complication of motor behavior as evidenced in the open field activity assessment. To further examine anxiety of IBO induced animals, other behavioral parameters were assessed including rearing, grooming, and head dipping. IBO induced animals engaged in more head dipping (Figure 7B) as ambulatory response, but there were no differences in rearing and grooming (data not shown). It is indeed reported that NR2A KO mice exhibit deficits in learning and memory performance and certain forms of synaptic plasticity, along with hypersensitivity to behavioral effects of antipsychotics (Kishimoto et al., 2001). RSV administration has been significantly reduced the behavioral abnormalities (Figure 7B) to a significance of *p* < 0.01.

IBO found to exhibit excitotoxicity either by oxidative stress-mediated cell death or as source-specific that might be the consequential event of calcium influx through NMDA receptor, where source specificity is reported to exhibit toxicity through nNOS activity increasing the production of nitric oxide levels inducing cell death (Di Biase et al., 2008; Soriano et al., 2008). The present study using IBO mediated excitotoxicity on NMDA receptors reflected an increase in nNOS expression that simultaneously increased nitric oxide levels as evidenced by measurement of nitrites (Figures 8A,B). Evidence suggests that impaired cholinergic signaling is a consequence of neuronal vulnerability to free radicals like nitric oxide mediated nitrites *in vivo* (Mattson, 1998). Moreover, nitric oxide is reported to alter ACh release (Guevara-Guzman et al., 1994) disrupting cholinergic transmission. This supported our findings on the increased activity of AChE in response to α7-nAChR expression, providing the strategies developed by cholinergic neurons during excitotoxicity. RSV exhibited a rescue effect decreasing AChE activity rejuvenating synaptic junctions of cholinergic neurons through its antioxidant activity. Further, reduction in nNOS and nitrite levels in IBO induced rats by RSV might have imparted a positive influence in reverting cholinergic abnormalities.

Antioxidant property of RSV was well-proved in the current observation, where RSV could normalize antioxidants like GPX activity (Figure 9A), GSH (Figure 9B) simultaneously reducing the levels of macromolecular damage decreasing protein carbonylation (Figure 9C), and Lipid peroxidation (Figure 9D). Glutathione (GSH) can serve as a neuromodulator/neurotransmitter that binds to NMDA receptors via its γ-glutamyl moiety (Janáky et al., 1999) and is thought to exert dual action as an agonist and antagonist on neuronal responses mediated by NMDA receptors in the brain (Aoyama et al., 2011). GSH is required for cell proliferation and neuronal differentiation (Poot et al., 1995). The observed reduction in GSH levels on IBO induced rat hippocampus indicates the rate of increase in oxidative stress. Oxidative stress is the major factor implicated in the pathogenesis of AD. GSH works as an electron donor for the reduction of H_2_O_2_ or other peroxidases catalyzed by GPx. The GPx levels were further studied to be significantly decreased on IBO induction. Numerous investigations have reported that RSV could directly scavenge various free radicals viz., superoxide, peroxyl, and hydroxyl radicals (Leonard et al., 2003; Bradamante et al., 2004; Schmatz et al., 2012). Our results clearly suggested that RSV administration has potentiated the increase in GPx activity and GSH levels that had rendered positive impact on reducing protein carbonylation and lipid peroxidation levels. Thus, RSV considered as an efficient drug target against IBO induced excitotoxicity preventing cell enhancing pyramidal neurons of the hippocampus and is reflected in the normal architecture of IBO induced rats.

**Figure:**
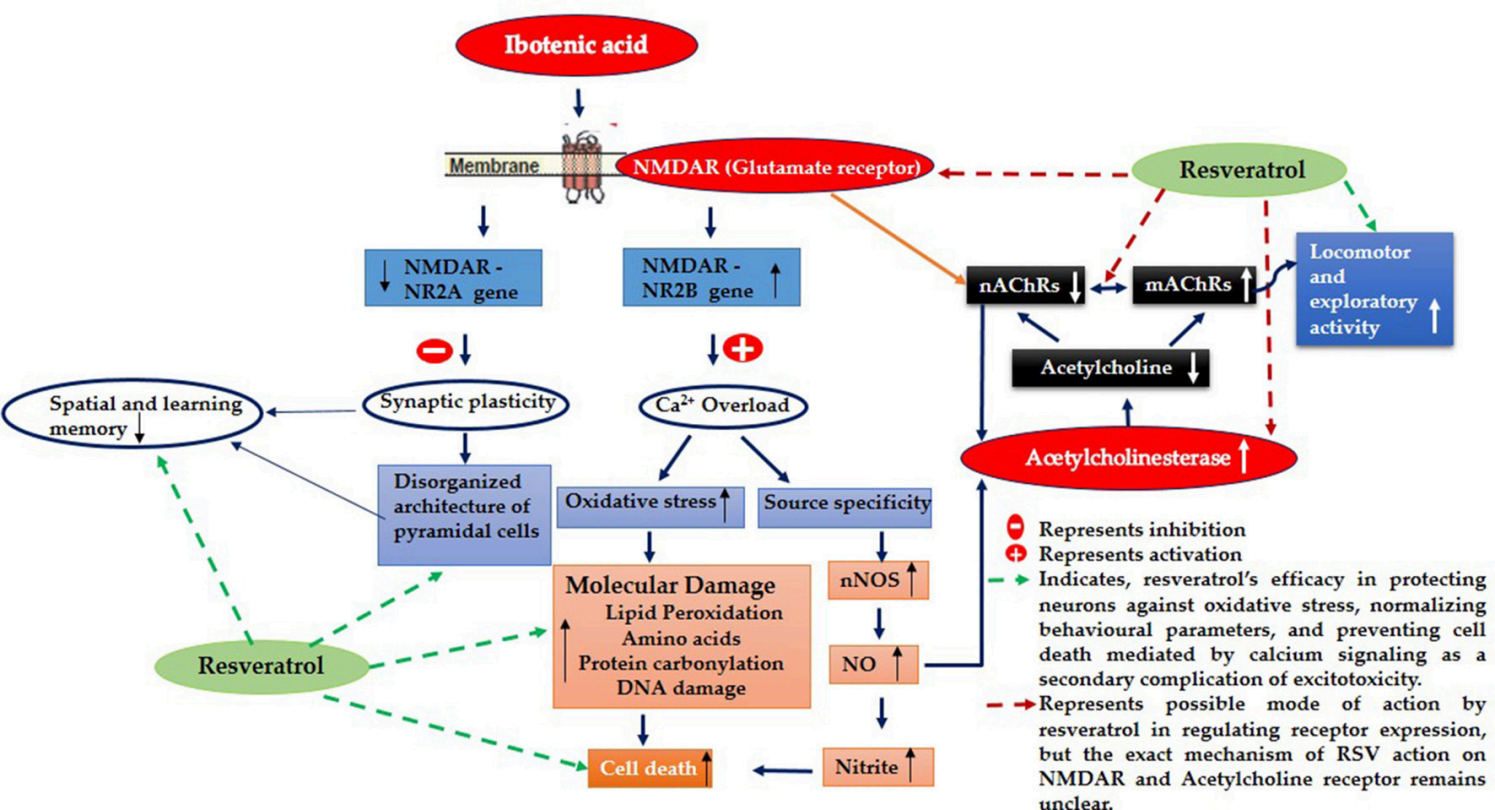


## Conclusion

The current study proved that IBO induction into hippocampal CA3 region mediated its toxicity through activation of NMDA subunits that negatively influenced cholinergic receptors resulting in memory impairment and defective locomotor behavior. While the mechanism behind alteration in cholinergic receptor still remains elusive. RSV exhibited protective role by normalizing the expression of glutamatergic and cholinergic receptors, maintaining homeostasis that may be due to its influence on SIRT1 *in vivo* as reported in our earlier studies. Further, the positive influence of RSV on IBO toxicity was evident through reduction in macromolecular damage, reduced morphological abnormalities and normal behavioral patterns proving its ability as potent neuroprotective compound. It is therefore could be considered as a promising agent to treat degenerating neuronal diseases accompanied by memory loss.

## Author contributions

CK and MA designed the research work; CK and KJ did Stereotaxic surgery for IBO induction; CK and SP performed Biochemical analysis, Behavioral analysis, and RT-PCR studies. CK and MA interpreted the data, and drafted the manuscript. All authors read and approved the final manuscript.

### Conflict of interest statement

The authors declare that the research was conducted in the absence of any commercial or financial relationships that could be construed as a potential conflict of interest.

## Abbreviations

AD: Alzheimer's disease
CA: Cornu ammonis
CNS, Central Nervous System, IBO: Ibotenic acid
RSV: Resveratrol
NMDA: N-methyl D-aspartate
NMDAR: N-methyl D-aspartate receptor
NR2A: N-methyl D-aspartate receptor 2A
NR2B: N-methyl D-aspartate receptor 2B
Ach: Acetylcholine
AChE: Acetylcholinesterase
α7-nAChR: α7-nicotinic acetylcholine receptor
GSH: Reduced glutathione
GPx: Glutathione peroxidase
MDA: Malondialdehyde
m1AChR: muscarinic acetylcholine receptor 1
nNOS: neuronal nitric oxide synthase
LTP: long term potentiation
LTD: long term depression
PCO: Protein carbonyl
RAM: Radial arm maze.
