# Supplementary material for: Intrahippocampal Administration of Ibotenic Acid Induced Cholinergic Dysfunction via NR2A/NR2B Expression: Implications of Resveratrol against Alzheimer Disease Pathophysiology
Source: Front Mol Neurosci. 2016 Apr 26;9:28. doi: 10.3389/fnmol.2016.00028 (PMC4844917; doi:10.3389/fnmol.2016.00028)
Supplement: Supplementary file 1 [file DataSheet1.DOCX]

***Supplementary Material***

**INTRAHIPPOCAMPAL ADMINISTRATION OF IBOTENIC ACID INDUCED CHOLINERGIC DYSFUNCTION *via* NR2A/NR2B EXPRESSION: IMPLICATIONS OF RESVERATROL AGAINST ALZHEIMER DISEASE PATHOPHYSIOLOGY**

**Chennakesavan Karthick^a^, Sabapathy Periyasamy^a^, Kesavan Swaminathan Jayachandran^b^, Muthuswamy Anusuyadevi ^a^ ***

^a^ Molecular Gerontology Laboratory, Department of Biochemistry (DST-FIST Sponsored), Bharathidasan University, Tiruchirappalli-620024, India.

^b^ Department of Bioinformatics, Bharathidasan University, Tiruchirappalli-620024, India.

* Corresponding author:

**Dr. M. Anusuyadevi**

Assistant Professor

Molecular Gerontology Laboratory,

Department of Biochemistry,

(DST-FIST Sponsored)

Bharathidasan University,

Tiruchirappalli-620024

Tamilnadu, India.

Tel.: +91 431 2407071 (Ext: 438); Fax: +91 431 2407045

E-mail address: [msanushyas2005@gmail.com](mailto:msanushyas2005@gmail.com)

**1. Supplementary Figure**

***********************Insert Supplementary Figure S1 about here** ********************

**Supplementary Figure S1 (a & b).** Diagrams of coronal rat brain section showing the location of microinjector tips (red arrow) in the hippocampus. Illustrations were adapted from the atlas of Paxinos and Watson, 6^th^ edition, 2007 (S1, a). The black arrow represents induction site at ventral portions of the CA3 region of hippocampus marked using trypan blue (S1, b).

**2. Supplementary Figure**

***********************Insert Supplementary Figure S2 about here** ********************

**Supplementary Figure S2**. Photomicrographs showing the effect of RSV on IBO induced in vivo toxicity in amygdala region of rat brain examined with H & E staining (magnification 40x)
