# Supplementary figures and images for: Intrahippocampal Administration of Ibotenic Acid Induced Cholinergic Dysfunction via NR2A/NR2B Expression: Implications of Resveratrol against Alzheimer Disease Pathophysiology
Source: Front Mol Neurosci. 2016 Apr 26;9:28. doi: 10.3389/fnmol.2016.00028 (PMC4844917; doi:10.3389/fnmol.2016.00028)

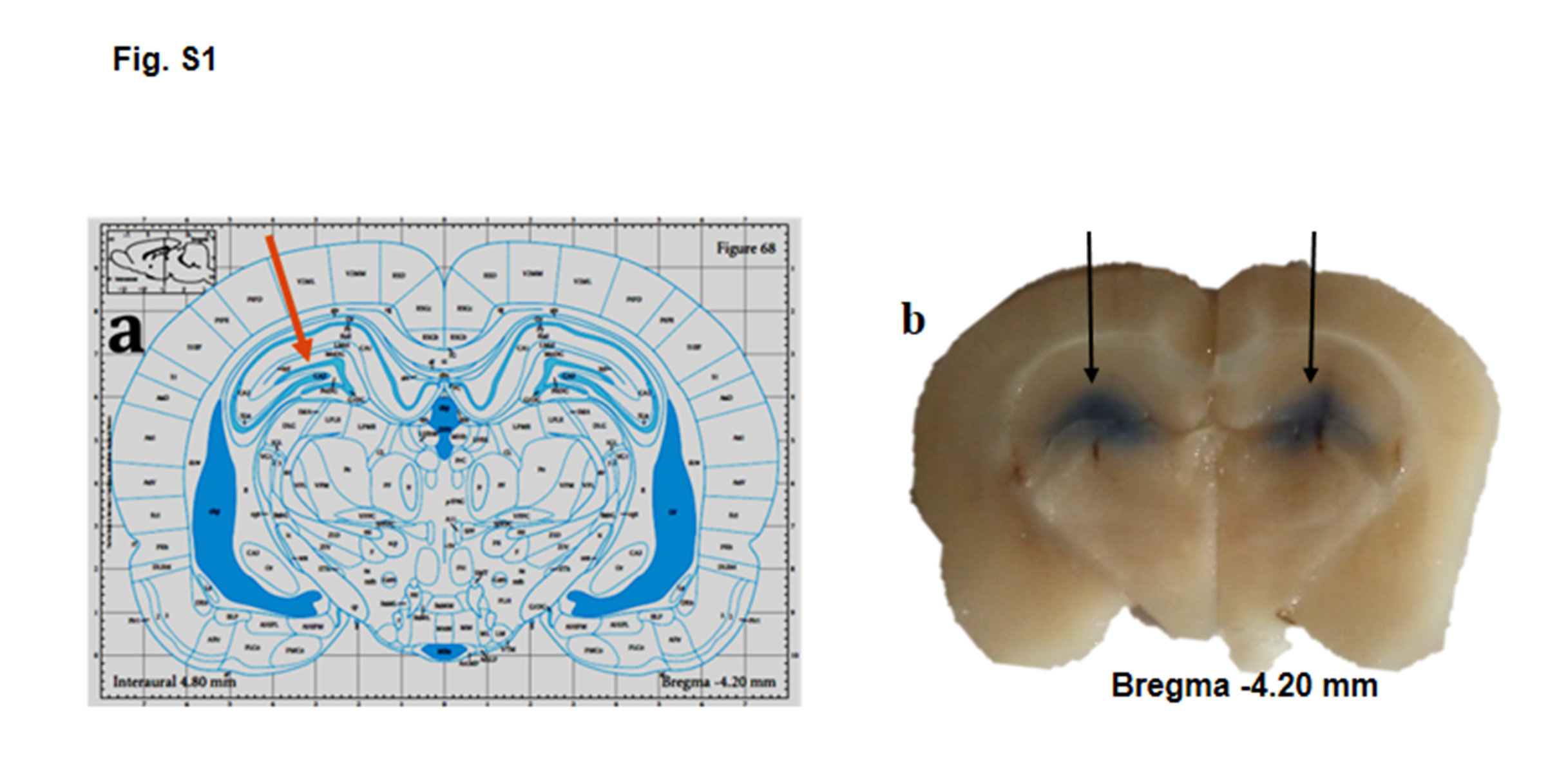

Supplement: Supplementary file 2 [file Image1.TIF]

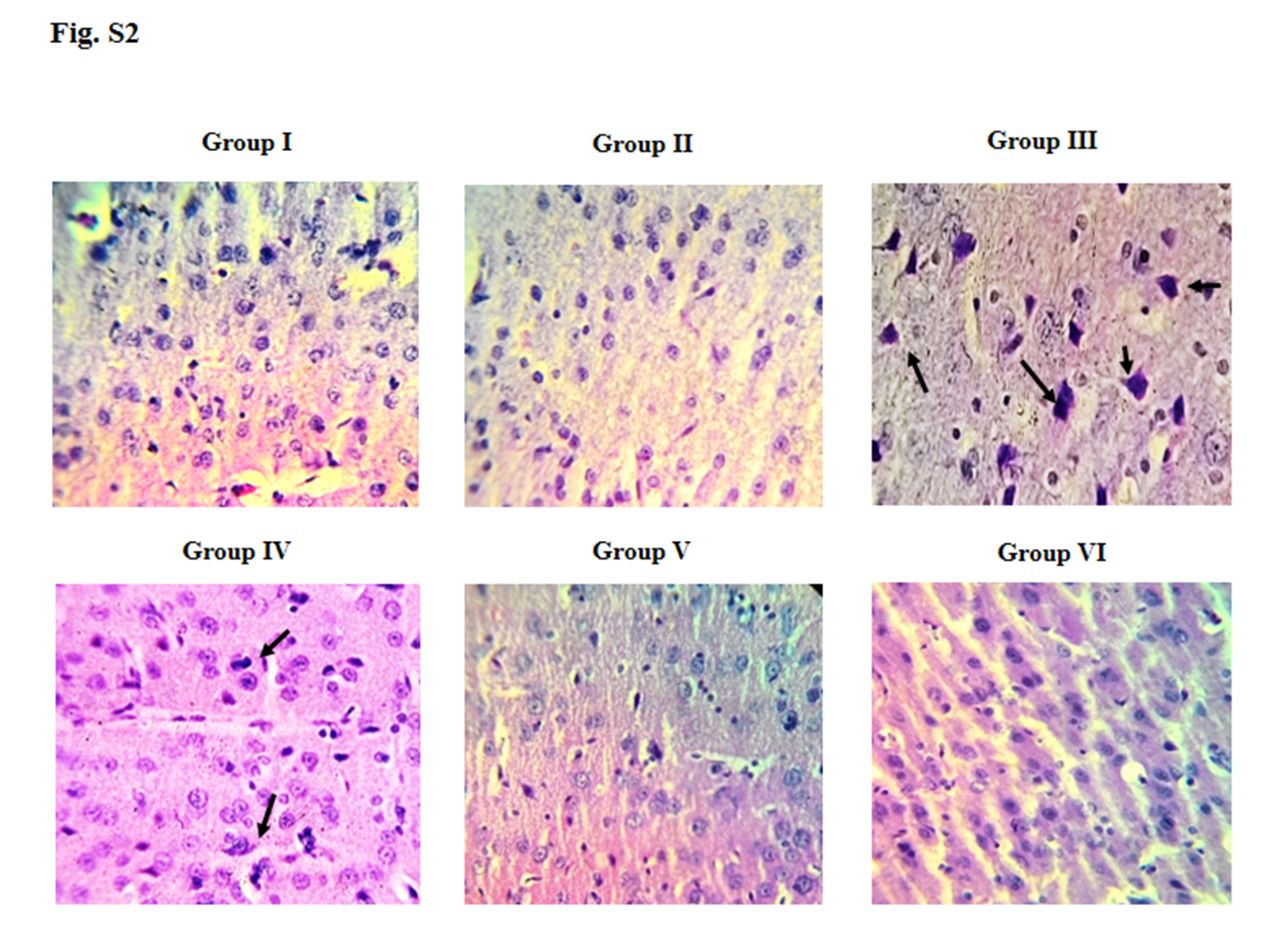

Supplement: Supplementary file 3 [file Image2.TIF]
